# A Biomimetic Biphasic Scaffold Consisting of Decellularized Cartilage and Decalcified Bone Matrixes for Osteochondral Defect Repair

**DOI:** 10.3389/fcell.2021.639006

**Published:** 2021-02-19

**Authors:** Runfeng Cao, Anqi Zhan, Zheng Ci, Cheng Wang, Yunlang She, Yong Xu, Kaiyan Xiao, Huitang Xia, Li Shen, Depeng Meng, Chang Chen

**Affiliations:** ^1^Department of Cardiothoracic Surgery, Shanghai Children’s Hospital, Shanghai Jiao Tong University, Shanghai, China; ^2^Department of Plastic and Reconstructive Surgery, Shanghai Ninth People’s Hospital, Shanghai Jiao Tong University School of Medicine, Shanghai Key Laboratory of Tissue Engineering, Shanghai, China; ^3^Research Institute of Plastic Surgery, Weifang Medical College, Shandong, China; ^4^Department of Orthopedics, Changzheng Hospital, Naval Medical University, Shanghai, China; ^5^Department of Thoracic Surgery, Shanghai Pulmonary Hospital, Tongji University School of Medicine, Shanghai, China

**Keywords:** biphasic scaffold, decellularized cartilage matrix, decalcified bone matrix, osteochondral repair, tissue engineering

## Abstract

It is challenging to develop a biphasic scaffold with biomimetic compositional, structural, and functional properties to achieve concomitant repair of both superficial cartilage and subchondral bone in osteochondral defects (OCDs). This study developed a biomimsubchondraletic biphasic scaffold for OCD repair via an iterative layered lyophilization technique that controlled the composition, substrate stiffness, and pore size in each phase of the scaffold. The biphasic scaffold consisted of a superficial decellularized cartilage matrix (DCM) and underlying decalcified bone matrix (DBM) with distinct but seamlessly integrated phases that mimicked the composition and structure of osteochondral tissue, in which the DCM phase had relative low stiffness and small pores (approximately 134 μm) and the DBM phase had relative higher stiffness and larger pores (approximately 336 μm). *In vitro* results indicated that the biphasic scaffold was biocompatible for bone morrow stem cells (BMSCs) adhesion and proliferation, and the superficial DCM phase promoted chondrogenic differentiation of BMSCs, as indicated by the up-regulation of cartilage-specific gene expression (ACAN, Collagen II, and SOX9) and sGAG secretion; whereas the DBM phase was inducive for osteogenic differentiation of BMSCs, as indicated by the up-regulation of bone-specific gene expression (Collagen I, OCN, and RUNX2) and ALP deposition. Furthermore, compared with the untreated control group, the biphasic scaffold significantly enhanced concomitant repair of superficial cartilage and underlying subchondral bone in a rabbit OCD model, as evidenced by the ICRS macroscopic and O’Driscoll histological assessments. Our results demonstrate that the biomimetic biphasic scaffold has a good osteochondral repair effect.

## Introduction

Osteochondral defects (OCDs) are a frequently occurring illness characterized by the concurrent injury of articular cartilage and subchondral bone tissue (Qiao et al., 2021). Currently, osteochondral autograft transplantations and decellularized osteochondral grafts are employed to treat OCDs (Richter et al., 2016; Wang et al., 2018). However, osteochondral autograft transplantations have insufficient sources and donor site mobility (Sherman et al., 2017). Although decellularized osteochondral grafts overcome the disadvantage of an insufficient source, there are still several limitations, such as poor integration between grafts and the surrounding normal tissue, and the compact cartilage layer severely restricting tissue remodeling, which greatly hamper their clinical outcomes (Farr et al., 2016). Therefore, an efficient therapeutic strategy is needed to treat OCD by restoring the intrinsic superficial cartilage and underlying bone in natural osteochondral tissue. Emerging tissue engineering strategies have provided such treatments with significant advantages compared with these traditional clinical treatments (Hu et al., 2020; Zhang et al., 2020).

At present, structural mimic biphasic osteochondral scaffolds consisting of synthetic or natural materials are widely used to repair OCDs, such as poly(lactide-co-glycolide) (Pan et al., 2015), bacterial cellulose (Zhu et al., 2018), and silicon-based bioceramic (Bunpetch et al., 2019). To achieve biological functions in separate structural phases, bioactive factors such as kartogenin and transforming growth factor-β are usually used for chondral phase (Mendes et al., 2018; Xuan et al., 2020), while bioactive molecules such as hydroxyapatite and bioactive factors such as bone morphogenetic protein-2 are generally incorporated to enhance osteoinduction in the bone phase (Betz et al., 2017; Xiao et al., 2019). Although incorporation of bioactive factors or molecules provides a regenerative microenvironment, they increase the fabrication cost of scaffolds and the release of bioactive substances is difficult to regulate (Aravamudhan et al., 2013; Santo et al., 2013). Hence, it is of great significance to fabricate scaffolds with intrinsic bioactivity for application in osteochondral tissue engineering.

Although those previously reports of biphasic scaffolds try to mimic the normal osteochondral tissue characterizations (Erickson et al., 2019; Lin et al., 2020a; Shang et al., 2020), the majority of materials that are used for OCD repair do not recapitulate the inherent extracellular matrix (ECM) in natural osteochondral tissue and are thus unable to rehabilitate the innate osteochondral structure and function (Pati et al., 2014). A decellularized cartilage matrix (DCM) and decalcified bone matrix (DBM) have inherent biological activities, native architectures, and excellent biocompatibility, which provide a desirable microenvironment for the regeneration of cartilage and bone tissue, respectively. Thus, DCMs and DBMs are regarded as ideal materials for cartilage and bone engineering. However, no reports on how to simultaneously apply these biomaterials to OCD repair are available. We speculated that a biphasic scaffold with a superficial DCM and underlying DBM would have the specific biological activities of the osteochondral phase. Fabricated biphasic scaffolds with a DCM and DBM not only closely resemble decellularized osteochondral grafts, but also solve integration and remodeling problems by an influx of stem cells and subsequent tissue regeneration.

**SCHEME 1:**
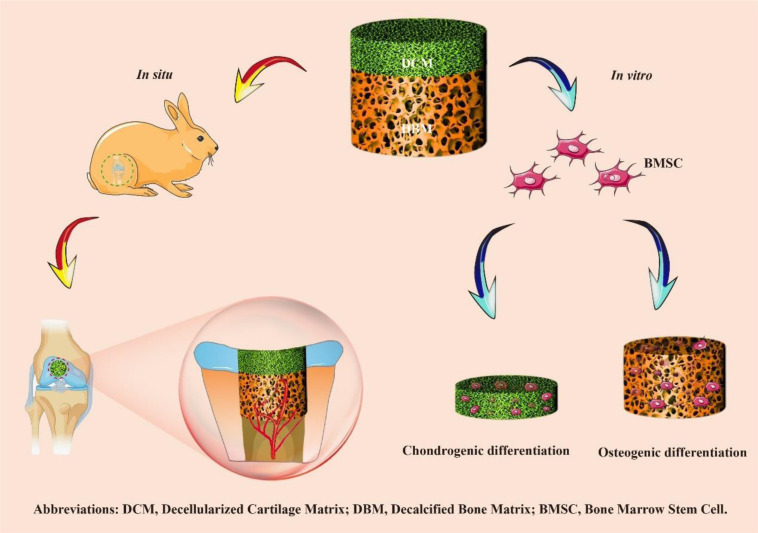
Overview of the experimental procedure. Briefly, a biomimetic biphasic scaffold consisting of a superficial DCM phase with relative low stiffness and small pores (approximately 134 μm) and an underlying DBM phase with relative higher stiffness and larger pores (approximately 336 μm) was fabricated by an iterative layered lyophilization technique. Additionally, the individual DCM and DBM layers were recolonized with BMSCs and cultured *in vitro* to evaluate chondrogenic and osteogenic differentiation. Furthermore, the biphasic DCM/DBM scaffold was used to evaluate the repair effect in a rabbit OCD model.

Here, a biomimetic biphasic scaffold consisting of a superficial DCM phase with relative low stiffness and small pores (approximately 134 μm) and an underlying DBM phase with relative higher stiffness and larger pores (approximately 336 μm) was fabricated by an iterative layered lyophilization technique. The biphasic scaffold with specific but seamlessly integrated phases mimicked the composition and structure of natural osteochondral tissue, which was biocompatible for bone morrow stem cells (BMSCs) adhesion and proliferation. Additionally, we investigated whether the relative low stiffness and small porosity of the superficial DCM phase promoted cartilage formation, while the inherent relative higher stiffness and large pores of the DBM phase induced bone regeneration *in vitro*. Finally, we evaluated whether the biphasic DCM/DBM scaffold could enhance *in situ* OCDs repair in rabbit (Scheme 1).

## Materials and Methods

### Biphasic DCM/DBM Scaffold Synthesis

Fresh bovine articular cartilage was obtained from a local slaughterhouse. After cooling with liquid nitrogen, the articular cartilage was ground into powder and decellularized in sterile 2% sodium dodecyl sulfate (Sigma) at 4°C for 4 h, washed in phosphate-buffered saline (PBS) for 1 h, digested in a 200 U/ml DNase I solution (Sigma) for 4 h, and then washed in PBS for 1 h (Xu et al., 2017). Four times of the above decellularization procedure were repeated to obtain the DCM sample.

Bovine cancellous bone was removed from soft tissues, cut, and shaped, followed by supercritical carbon dioxide degreasing and freeze drying to obtain a cancellous bone sample. The cancellous bone was decalcified in a 0.5 mol/L hydrochloric acid solution at a weight ratio of 20:1 in a conical flask with shaking at 25°C at a rotation speed of 120 rpm for 48 h. The decalcified cancellous bone sample was washed in an ultrasonic cleaner for 2 h, which was repeated five times, lyophilized, and then sterilized by gamma irradiation at a dose of 25 KGy to obtain the DBM sample.

The DCM was suspended in deionized water (2% w/v) and poured into a cylinder-shaped silicone mold (diameter: 4 mm; height: 4 mm) to 1 mm in height, and then frozen at -80°C for 2 h. Thereafter, the DBM with a height of 3 mm was stacked onto the DCM scaffold inside the mold, and frozen at -80°C for 2 h. Thereafter, the biphasic DCM/DBM scaffold was lyophilized for 24 h, crosslinked with a carbodiimide solution (48 mM EDC and 6 mM NHS in 50-mM MES buffer; pH = 5.5) at 4°C for 24 h, and disinfected by ethylene oxide. DCM (diameter: 4 mm; height: 1 mm), DBM (diameter: 4 mm; height: 1 mm), and biphasic DCM/DBM (diameter: 4 mm; height: 4 mm) were prepared for the following experiment use.

### Characterization of the Biphasic Scaffold

The critical points of DCM, DBM and biphasic scaffolds were dried and scoped by scanning electron microscopy (SEM). The pore diameter in each scaffold was reckoned using ImageJ.

A Nicolet-Nexus 670 FTIR spectrometer (Thermo Fisher Scientific, Waltham, MA, United States) was used to obtain FTIR spectra of the DCM, DBM, DCM/DCM scaffold over the range of 200–4000 cm^–1^ at a scanning resolution of 2 cm^–1^.

A liquid displacement method was used to determine the porosity of the scaffold. The original volume of ethanol was designated as V_0_, the volume after the scaffold was immersed in ethanol for 5 min was designated as V_1_, and the residual volume after taking out the wet scaffold was designated as V_2_. The scaffold porosity was reckoned by the equation: (V_0_−V_2_)/(V_1_−V_2_) × 100% (Li et al., 2020).

To test the mechanical properties of the DCM and DBM scaffolds, cylindrical-shaped samples (diameter: 4 mm; height: 2 mm) were compressed to 30% strain at a speed of 3 mm/min and its compressive modulus was reckoned according to the initial stress-strain curve (Xu et al., 2020b).

The dry weight of each scaffold was initial weighed as W_0_. Then, the scaffold was immersed in deionized water for 2, 4, 6, and 8 min, respectively, and weighed again as W_1_. The water absorption rate of the scaffold was reckoned by the equation: (W_1_−W_0_)/W_0_ × 100% (Xu et al., 2020a).

The protein adsorption capacity of the scaffolds was determined according to the BCA protein assay kit (Beyotime, China) as previously described methods (Jee et al., 2004).

### Biocompatibility Testing

This experiment was approved by the Animal Care and Experiment Committee of the Shanghai Children’s Hospital. BMSCs were cultured as a conventional method (Xu et al., 2019). To test the biocompatibility of the scaffolds, both DCM and DBM scaffolds were implanted with BMSCs at passage two (5 × 10^5^ cells/mL) and cultured in Dulbecco’s modified Eagle’s media (DMEM) (Gibco) supplemented with 10% fetal bovine serum (FBS) (Gibco) and 1% penicillin-streptomycin (routine culture media). BMSCs cultured in the routine culture media were served as control group. Cells viability in the scaffolds were determined using a Live and dead cell viability assay (Invitrogen, United States) and a Cell Counting Kit-8 (CCK-8) (Dojindo, Japan) after *in vitro* culture for 1, 5, and 9 days.

### Cell Adhesion Rate

The total BMSCs seeded on to the scaffold were counted as N_0_. After incubation for 24 h, BMSCs in the culture dish were collected and counted as N_1_. The cell adhesion rates of the scaffolds were reckoned by the equation: (N_0_−N_1_)/N_0_ × 100% (Zhang et al., 2019).

### Chondrogenic and Osteogenic Differentiation

Bone morrow stem cells at passage two (2 × 10^7^ cells/mL) were seeded uniformly onto DCM and DBM scaffolds. After incubation for 4 h, the BMSC-laden DCM and DBM scaffolds were cultured in the routine culture media. After 7 or 14 days of maintenance under *in vitro* conditions, the gene expression of BMSCs in each scaffold group was determined by real-time polymerase chain reaction (qPCR) examination. BMSCs cultured alone in regular culture medium were used as the control group. Total RNA was extracted using Trizol reagent (TaKaRa, Shiga, Japan) and reverse transcribed into cDNA with a PrimeScript RT reagent kit (TaKaRa, Shiga, Japan). qPCR was conducted using a real-time qPCR system (LightCycler 480 II; Roche Diagnostics Ltd., Shanghai, China) to evaluate the expression of cartilage-related markers ACAN, Collagen II, and SOX9 with housekeeping gene β-actin for normalization. The data were analyzed using the ΔΔCt method to determine relative gene expression. Primers were obtained from Sangon Biotech (Shanghai, China). Primer sequences are listed in Supplementary Table 1.

### *In situ* Surgical Implantation

Twelve healthy 4-month-old New Zealand white rabbits (Approximately 2.5–3 kg) were averagely divided into two groups: a biphasic DCM/DBM scaffold group and untreated group (no scaffold) as the control. Sutera (0.3 mL/kg) were used to anesthetize the rabbits and OCDs (diameter: 4 mm; height: 4 mm) were drilled on the trochlear groove of rabbit knee joints. The OCD samples were harvested at 6- and 12-weeks post-operatively and evaluated using the International Cartilage Repair Society (ICRS) macroscopic score as listed in Supplementary Table 2.

### Histological Observation

Took samples and fixed these samples in 4% paraformaldehyde, in which the OCD samples were further decalcified in a 10% ethylenediaminetetraacetate dihydrate solution for 3 weeks. The paraffin section was prepared and then hematoxylin and eosin (H&E) staining were carried out to evaluate the tissue structure, Toluidine blue and Safranin-O and Fast Green (SO/FG) were performed to evaluate cartilage and bone ECM deposition. Expression of type II and I collagens was stained to confirm the cartilage-specific and bone-specific phenotype as described previously (Zhou et al., 2020). OCD sections were further accessed via the O’Driscoll histological score as listed in Supplementary Table 3.

### Micro-Computed Tomography (micro-CT) Observation

Osteochondral defect samples were evaluated with a micro-CT scanner (μCT-80, Scanco Medical, Switzerland) in high-resolution scanning mode, and bone defects in harvested samples were visualized using 3D isosurface rendering at 6- and 12-weeks spatio-temporally and in bilayer sets. Micro-CT was applied to access trabecular thickness (Tb.Th) and the percentage of neo-bone volume relative to tissue volume (BV/TV).

### Statistical Analyses

Statistical analyses were performed via Origin 8.0 software. For the two independent samples, we used *t* test. For the repeated measurement data from different groups, we used Two-way Repeated Measures ANOVA. *P* < 0.05 was deemed statistically significant. Values are reported as the mean ± standard deviation from five specimens.

## Results and Discussion

### Morphology, Porosity, Mechanical Properties, and Absorption Capacity of DCM and DBM Scaffolds

Osteochondral tissue has a distinct structure consisting of superficial cartilage and underlying subchondral bone, integrates well with each other to achieve optimal weight-bearing and joint mobility functions. With a natural ECM architecture and inherent biological activity, DCM and DBM are considered to be ideal scaffolds for cartilage and bone regeneration, respectively. Considering that the osteochondral structure has two distinct phases, we employed an iterative layered lyophilization technique to prepare a biomimetic and biphasic DCM/DBM scaffold with two different substrates to simulate the biphasic composition of natural osteochondral tissue. We successfully prepared the biomimetic biphasic scaffold in a cost-effectiveness method, however, considering the underlying DBM scaffold comes from the cancellous bone, the pore size and stiffness is hard to be modulated (Liese et al., 2013; Man et al., 2016).

**FIGURE 1:**
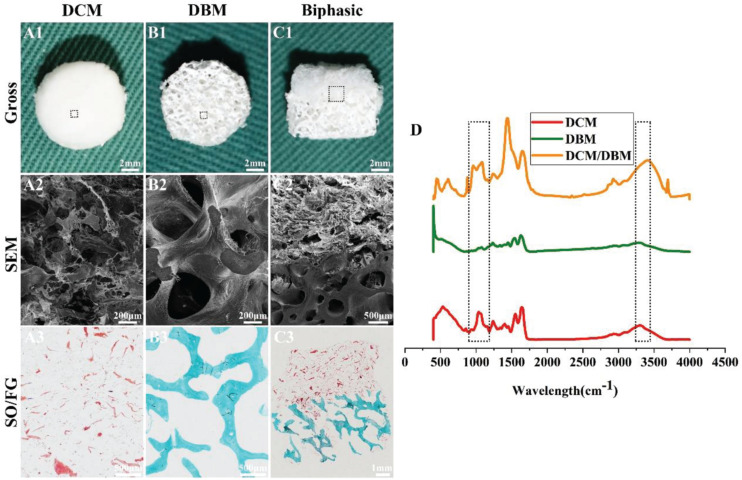
Morphology and FTIR examinations of DCM, DBM, and biphasic DCM/DBM scaffolds. Gross images **(A1–C1)** of DCM, DBM, and biphasic DCM/DBM scaffolds; SEM images **(A2–C2)** of DCM, DBM, and biphasic DCM/DBM scaffolds; SO/FG staining images **(A3–C3)** of DCM, DBM, and biphasic DCM/DBM scaffolds. FTIR analysis of DCM, DBM, and biphasic DCM/DBM scaffolds **(D)**.

**FIGURE 2:**
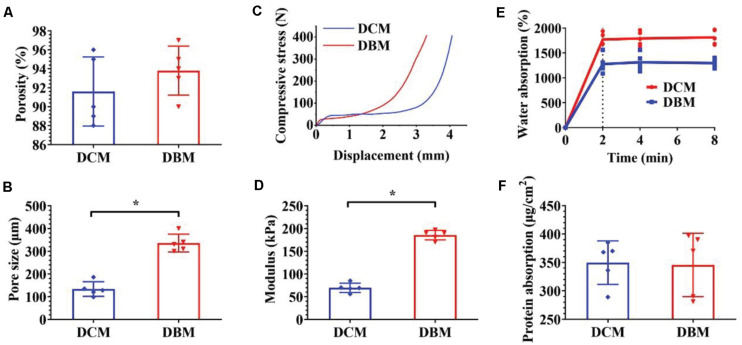
Characterization of individual DCM and DBM scaffolds. The pore size **(A)**, porosity **(B)**, compressive stress **(C)**, Young’s modulus **(D)**, water absorption **(E)**, and protein absorption **(F)** of individual DCM and DBM scaffolds. ^∗^*p* < 0.05.

As shown in Figure 1, integration of the DCM and DBM scaffolds yielded the biphasic DCM/DBM scaffold with the features of each scaffold type and no apparent gap between them as evidenced by gross, SEM, and HE staining images, as well as FTIR analysis (Figure 1D), which confirmed seamless integration between each individual layer by the iterative layering process. The stable integration in the biphasic scaffold would facilitate layer-specific ECM formation including cartilage and bone. Additionally, DCM, DBM, and biphasic DCM/DBM scaffolds exhibited a white appearance with apparently porous sponge structures (Figures 1A1–C1). Further, SEM images (Figures 1A2–C2) and SO/FG staining images (Figures 1A3–C3) also confirmed that all those scaffolds possessed a high degree of pore interconnectivity with homogeneous pore structure. Notably, the SO/FG staining confirmed that the superficial layer was cartilage-specific ECM as evidenced by positive SO staining and the underlying layer was bone-specific ECM as evidenced by positive FG staining. Additionally, quantitative analysis revealed that both DCM and DBM scaffolds exhibited high levels of porosity (>90%) (Figure 2A), which were favorable for host BMSC infiltration, nutrition exchange, and matrix secretion.

The pore size of a scaffold has been proved to regulate the differentiation direction and matrix deposition of BMSCs (Murphy et al., 2010; Levingstone et al., 2014). Additionally, the attachment and infiltration of cells could also be significantly affected by the scaffold pore size. Small pore sizes of approximately 100 μm facilitate chondrogenesis, whereas large pore sizes that exceed 300 μm promote osteogenesis (Karageorgiou and Kaplan, 2005; Gupte et al., 2018). The underlying mechanism is that small pores facilitate the induction of cartilage formation under hypoxic conditions, while large pores promote capillary proliferation and bone formation (Zhou et al., 2020). Additionally, on small islands where BMSCs adopted a rounded morphology, chondrogenesis is predominant, while on larger islands where BMSCs adopted a spread morphology, osteogenesis is favored (McBeath et al., 2004). The concentration of scaffold materials has a tremendous effect on the pore size in freeze-dried scaffolds in which the mean pore size decreases with increases DCM/DBM concentrations (Rowland et al., 2016). In the current study, by adjusting the DCM concentration, we customized the porous DCM scaffold with small pores (134 ± 28.8 μm) for chondrogenic differentiation, whereas the DBM scaffold showed an inherent porous structure with larger pores (336 ± 34.9 μm) for osteogenic differentiation (Figure 2B). Next, we prepared a biphasic DCM/DBM scaffold by an iterative layering freeze-drying technique that enabled control of the pore size within each layer of the biphasic scaffold.

Mechanical properties are also essential to design tissue engineering scaffolds. BMSC differentiation has been connected to the mechanical properties of the underlying scaffold, which affect mechanoreceptors and ultimately differentiation along specific lineages in response to these biomechanical cues (Her et al., 2013; Xue et al., 2013). Previous studies have suggested that BMSCs tended to chondrogenic differentiation on soft scaffolds and osteogenic differentiation on stiff scaffolds (Murphy et al., 2012; Olivares-Navarrete et al., 2017; Wu et al., 2018). The iterative layering process allowed tailoring the mechanical properties of each layer in the biphasic scaffold. Figures 2C,D show that the DCM and DBM scaffolds had different stress-strain curves and the DBM scaffold had a higher compressive modulus compared with the DCM scaffold. We speculated that BMSCs on the DCM scaffold with a lower compressive modulus would have elevated expression of ACAN, SOX9, and collagen II as well as GAG content, whereas BMSCs on DBM the scaffold with a relatively high compressive modulus may be able to differentiate into mature osteoblasts.

The adsorption capacity of scaffolds may also positively affect tissue regeneration (Li et al., 2020; Lin et al., 2020b). Deficient repair of an OCD is partly due to the insufficient amount of BMSCs released by the subchondral bone marrow and homing them to the defect. Additionally, load-bearing forces and fluid movement may physically prevent BMSCs from proliferating in the defect zone and hamper nutrition to sustain where they are required (Gomoll, 2012). Hence, scaffolds with an excellent water absorption rate facilitate cell attachment and desirable protein absorption efficiency promotes nutrition retention. Our results indicated that both the DCM and DBM exhibited satisfactory water and protein absorption capacities (Figures 2E,F). These favorable factors indicated that both DCM and DBM scaffolds could facilitate BMSC attachment and proliferation.

### Cell Viability and Adhesion Rate of DCM and DBM Scaffolds

**FIGURE 3:**
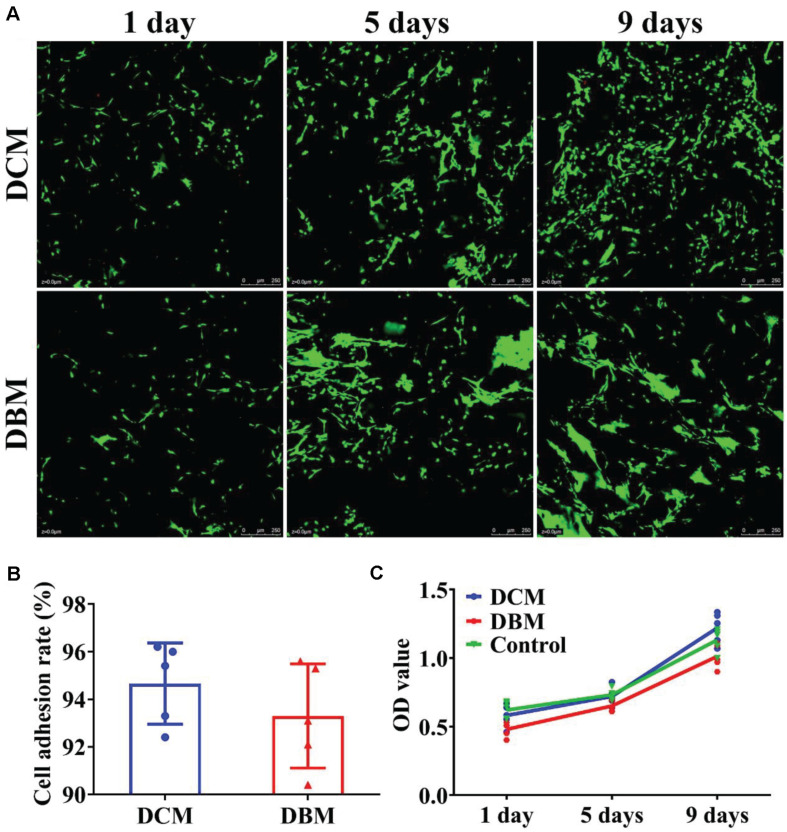
Biocompatibility evaluation of individual DCM and DBM scaffolds. Live and dead staining **(A)**, cell adhesion rate **(B)**, and cell proliferation assay **(C)** in individual DCM and DBM scaffolds.

Scaffolds with satisfactory cytocompatibility and an adhesion rate are highly desirable for OCD tissue engineering. Both the DCM and DBM are native tissue-derived scaffolds and biocompatible (Tan et al., 2009; Chen et al., 2020). Both DCM and DBM scaffolds were seeded with BMSCs to evaluate their potential as scaffolds for osteochondral engineering. BMSCs survived and proliferated well on both DCM and DBM scaffolds at 1–9 days after cell seeding, as indicated by Live and dead staining images (Figure 3A) and was further validated by the CCK-8 cell proliferation assay (Figure 3C). Additionally, both DCM and DBM scaffolds showed comparable high adhesion rates (Figure 3B), which may be positive affected by their satisfactory absorption capacity as described above. The ability of BMSCs to adhere and proliferate on the scaffolds as evidenced by the *in vitro* assessments confirmed the biocompatibility of both DCM and DBM scaffolds. Little difference in the cell number was observed between DCM and DBM scaffolds, which indicated that the procedure to fabricate both porous DCM and DBM scaffolds do not affect their biocompatibilities. Homogenous cellular distributions on both DCM and DBM scaffolds were demonstrated *in vitro*, which indicated that both scaffolds had the potential to allow host BMSCs to distributed uniformly on biphasic DCM/DBM scaffolds after *in situ* implantation.

### *In vitro* Chondrogenic and Osteogenic Differentiation on DCM and DBM Scaffolds

**FIGURE 4:**
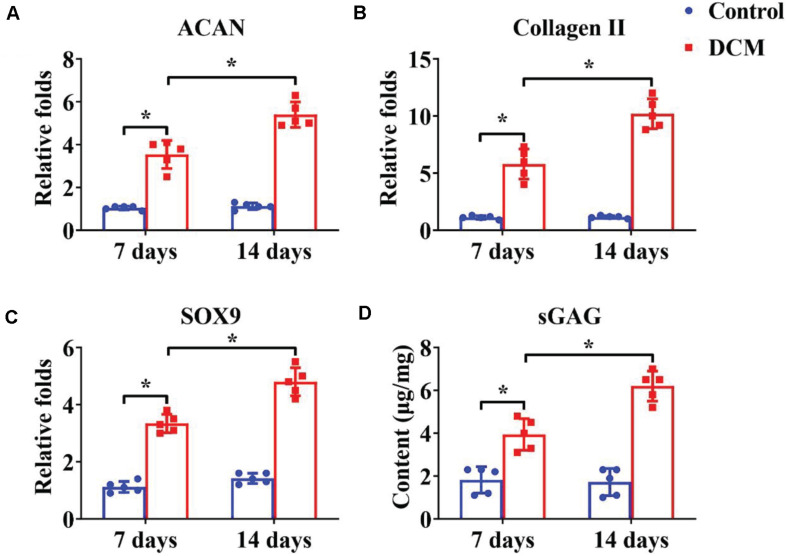
Chondrogenic differentiation of BMSCs cultured on the DCM scaffold for 7- and 14-days *in vitro* culture. Chondrogenic-related gene expression of ACAN **(A)**, collagen II **(B)**, and SOX9 **(C)**. GAG content of BMSCs on the DCM scaffold **(D)**. ^∗^*p* < 0.05.

**FIGURE 5:**
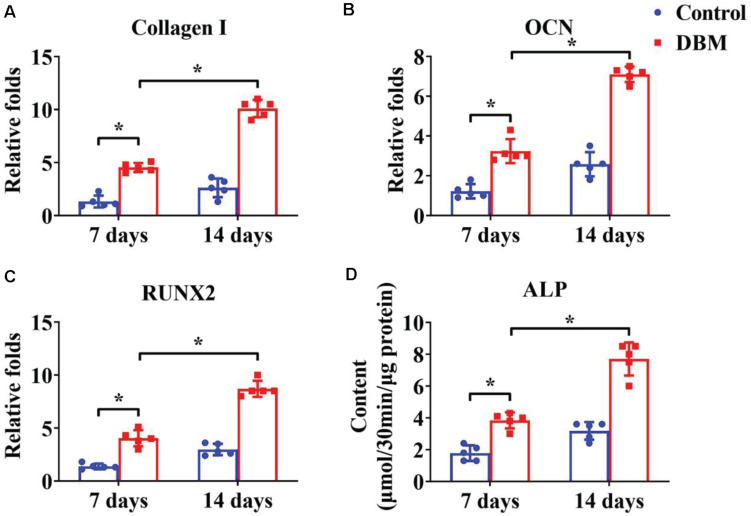
Osteogenic differentiation of BMSCs cultured on the DBM scaffold for 7- and 14-days *in vitro* culture. Osteogenic-related gene expression of collagen I **(A)**, OCN **(B)**, and RUNX2 **(C)**. ALP content of BMSCs on the DBM scaffold **(D)**. ^∗^*p* < 0.05.

Appropriate processing of decellularized tissue preserves the biochemical, microstructure, and inductive properties of the natural ECM, which promotes *in vitro* generation of site-specific functional tissue (Lee et al., 2018). BMSCs have a high ability to expand and can differentiate into chondrogenic and osteogenic lineages. Many factors influence BMSC differentiation, such as mechanical properties, chemical cues, and biological cues. Extensive studies have demonstrated that a DCM provides a natural chondrogenic microenvironment for BMSCs (Xue et al., 2012), whereas a DBM maintains inherent biological cues for osteogenesis of BMSCs (Mattioli-Belmonte et al., 2019). Our results revealed that the DCM scaffold had obviously higher expression of cartilage-specific genes (Figures 4A–C), including ACAN, collagen II, and SOX9, and apparently promote a cartilage-specific ECM by sGAG secretion (Figure 4D) compared with the control group. In a similar fashion, the DBM scaffold displayed high expression of bone-specific genes (Figures 5A–C) including collagen I, OCN, RUNX2, and a noticeably enhanced bone-specific ECM by ALP deposition (Figure 5D). The underlying mechanisms of the DCM scaffold promoting chondrogenic differentiation may be related to the retained structural and functional proteins of the cartilage-specific ECM, small pore size, and soft stiffness, and the DBM scaffold inducing osteogenic differentiation could be associated with its bone-specific ECM with inherent biological cues, large pore size, and rigid stiffness.

### *In situ* Regeneration of OCDs by the Biphasic DCM/DBM Scaffold

**FIGURE 6:**
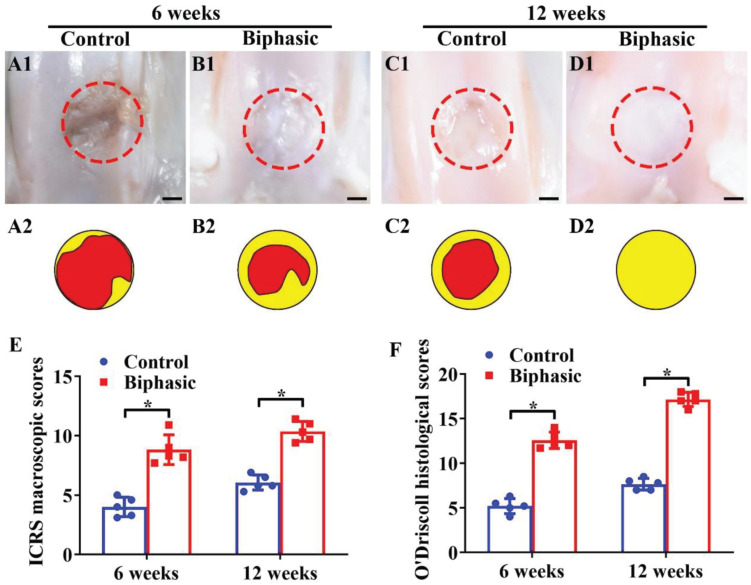
Microscopic and quantitative evaluations of repaired defects in control and biphasic groups. Gross view **(A1–D1)** and its corresponding schematic **(A2–D2)** images of samples in control and biphasic groups. In the schematic images, yellow circles denote the defect border immediately after the operation and red circles outline the edge of the defect at 6- and 12-weeks post-operatively. ICRS macroscopic scores of the samples in control and biphasic groups **(E)**. O’Driscoll histological scores of the samples in control and biphasic groups **(F)**. Scale bars = 2 mm.^∗^*p* < 0.05.

The *in situ* regeneration potential of the biphasic DCM/DBM scaffold with satisfactory *in vitro* chondrogenic and osteogenic efficacies was investigated in a rabbit OCD model. In order to access the feasibility of osteochondral repair *in situ*, the biphasic DCM/DBM scaffold was implanted into rabbit OCDs model. At 6 and 12 weeks post-implantation, the gross image of the osteochondral tissues indicted that the biphasic group had an apparently superior repair efficacy compared with the untreated control group (Figures 6A1–D1, A2–D2). Additionally, quantitative analyses of ICRS macroscopic and O’Driscoll histological scores confirmed that the biphasic group outperformed the control group (Figures 6E,F). Notably, complete filling of the defect with white cartilage-like tissue was displayed in the biphasic group at 12 weeks, whereas a clear unrepaired defect with thin fibrous tissue was still observed in the control group.

**FIGURE 7:**
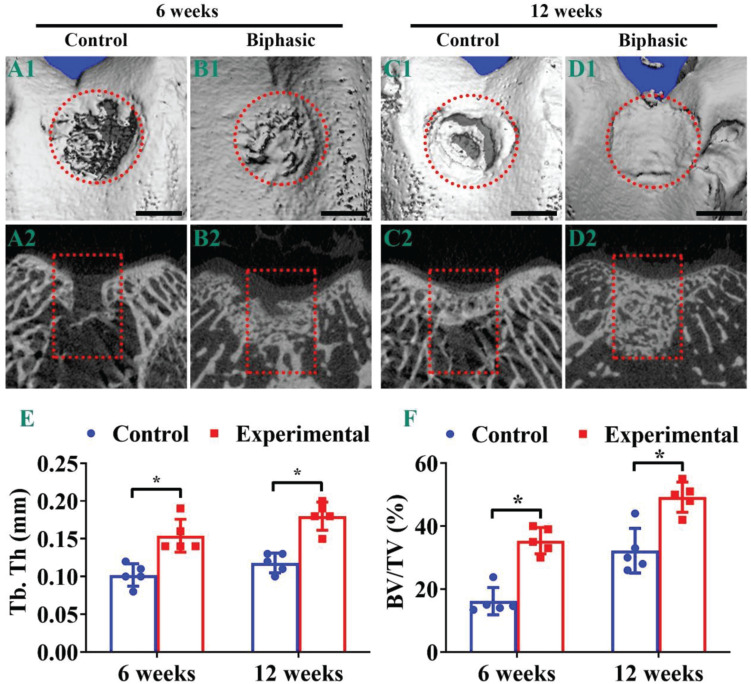
Micro-CT analysis of the samples in control and biphasic groups. Three-dimensional **(A1–D1)** and two-dimensional **(A2–D2)** images of the repaired defect in control and biphasic groups post-operatively. Quantitative data of Tb. Th in the repaired defect post-operatively **(E)**. Quantitative data of BV/TV in the repaired defect post-operatively **(F)**. OCDs are outlined by red dotted circles and rectangles. Scale bars = 2 mm. ^∗^*p* < 0.05.

Micro-CT observations were used to further evaluate the repair effect of OCDs post-implantation. Evidently, the biphasic group showed a better OCDs therapeutic effect compared with the control group, whereas the defects in control and biphasic groups presented an increasing recover trend from 6 (Figures 7A1,B1,A2,B2) to 12 weeks (Figures 7C1,D1,C2,D2). Additionally, the OCDs samples displayed a virtually complete OCD repair in the biphasic group at 12 weeks post-implantation, whereas the control group remained a large hollow at the defects (Figures 7A1–D1). The two-dimensional features of the OCDs showed that the control group still had a large area of non-regenerating subchondral bone in the defect, whereas the biphasic group had almost complete subchondral bone reconstruction in the defect. We also quantified the bone histomorphometric parameters of the neo-bone tissue in the region of interest. The mean Tb.Th (Figure 7E) and the BV/TV (Figure 7F) were much higher in the biphasic group compared with those in the control group after 6- and 12-weeks, indicating that the biphasic scaffold plays a considerable positive effect on promoting subchondral bone repair.

**FIGURE 8:**
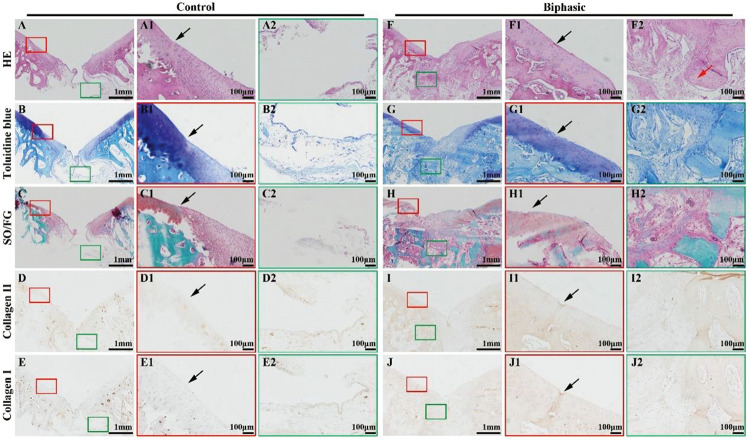
Histological evaluations of the samples in control and biphasic groups at 6 weeks post-operatively. HE staining images of control **(A–A2)** and biphasic **(F–F2)** groups. Toluidine blue staining images of control **(B–B2)** and biphasic **(G–G2)** groups. SO/FG staining images of control **(C–C2)** and biphasic **(H–H2)** groups. Collagen II immunohistochemical staining images of control **(D–D2)** and biphasic **(I–I2)** groups. Collagen I immunohistochemical staining images of control **(E–E2)** and biphasic **(J–J2)** groups. The black arrows separate native cartilage and neocartilage. The red arrow indicate neo-bone matrix.

**FIGURE 9:**
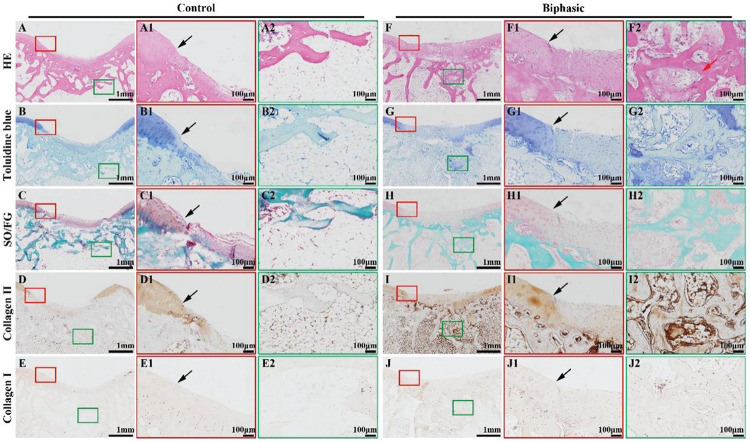
Histological evaluations of the samples in control and biphasic groups at 12 weeks post-operatively. HE staining images of control **(A–A2)** and biphasic **(F–F2)** groups. Toluidine blue staining images of control **(B–B2)** and biphasic **(G–G2)** groups. SO/FG staining images of control **(C–C2)** and biphasic **(H–H2)** groups. Collagen II immunohistochemical staining images of control **(D–D2)** and biphasic **(I–I2)** groups. Collagen I immunohistochemical staining images of control **(E–E2)** and biphasic **(J–J2)** groups. The black arrows separate native cartilage and neocartilage. The red arrow indicate neo-bone matrix.

Histological images, including HE, toluidine blue, SO/FG, and immunohistochemical collagen II and I staining, showed significant improvement in the repair of OCDs in the biphasic groups at 6 (Figure 8) and 12 (Figure 9) weeks post-implantation, as evidenced by a nice interface with excellent healing between the neo-cartilage tissue and its adjacent normal cartilage and sufficient regenerated trabecular bone presented over the biphasic scaffold area. In the contrast, an evidently depressed condition of the regenerated tissues with cavern in superficial neo-cartilage tissue and few underlying trabecular bones regeneration in the control group.

The underlying mechanisms of the satisfactory reparative efficacy are as follows. After implantation of compositional and structural biphasic scaffolds, the excellent absorption capacity ensures enrichment of stem cells derived from bone marrow. Then, with the inherent tissue-specific chemical composition and gradient porous structure, the biphasic scaffold provides a suitable niche for stem cell differentiation, which finally achieves cartilage and bone tissue regeneration. In contrast, because of the lack of stem cell adhesion sites and regenerative environment in the control group, the regeneration of osteochondral tissue was limited. Compared with concurrently adopted autologous and allogenic osteochondral implants, the biphasic DCM/DBM scaffold retains the chemical composition and has a tissue-remolding ability, which indicates that the biphasic DCM/DBM scaffold may be an ideal scaffold to replace osteochondral grafts for OCD tissue engineering.

## Conclusion

The current study fabricated a biomimetic biphasic DCM/DBM scaffold for OCD repair, which contains a superficial cartilaginous layer and underlying bone layer to mimic the inherent gradient structure of normal osteochondral tissue, with advantages in terms of the regeneration environment. The tissue-specific chemical composition coordinates with the gradient porosity and mechanical properties of biphasic scaffolds to promote space-dependent differentiation of stem cells by providing a tissue-specific environment as evidenced by the *in vitro* differentiation of stem cells and *in situ* OCD repair. Due to the composition of DCM/DBM scaffolds have been applied in clinical practice and well-studied, therefore the fabricated DCM/DBM scaffolds will take less time to achieve clinical application and translation. The current study provides a highly biomimetic scaffold composed with well-studied native-derived biomaterials for osteochondral tissue engineering in the future.

## Data Availability Statement

The raw data supporting the conclusions of this article will be made available by the authors, without undue reservation.

## Ethics Statement

This project was approved by the Shanghai Children’s Hospital Ethics Committee.

## Author Contributions

RC and AZ: scaffolds design. ZC and CW: culture cells. YS and YX: animal operation. LS and KX: SEM test. HX: review. DM and CC: funding acquisition and review and editing. All authors contributed to the article and approved the submitted version.

## Conflict of Interest

The authors declare that the research was conducted in the absence of any commercial or financial relationships that could be construed as a potential conflict of interest.
