# Supplementary material for: A Biomimetic Biphasic Scaffold Consisting of Decellularized Cartilage and Decalcified Bone Matrixes for Osteochondral Defect Repair
Source: Front Cell Dev Biol. 2021 Feb 19;9:639006. doi: 10.3389/fcell.2021.639006 (PMC7933472; doi:10.3389/fcell.2021.639006)
Supplement: Supplementary file 1 [file Table_1.DOCX]

**Supplemental Materials**

**A biomimetic biphasic scaffold consisting of decellularized cartilage and decalcified bone matrixes for osteochondral defect repair**

**Runfeng Cao^a,e,1^, Anqi Zhan^d,e,1^, Zheng Ci^d,e,1^, Cheng Wang^c^, Yunlang She^b^, Yong Xu^b^, Li Shen^a^, Kaiyan Xiao^e^, Huitang Xia^d,e,*^, Depeng Meng^c,*^, Chang Chen^b,*^**

^a^ Department of Cardiothoracic Surgery, Shanghai Children's Hospital, Shanghai Jiao Tong University, Shanghai, China

^b^ Department of Thoracic Surgery, Shanghai Pulmonary Hospital, Tongji University School of Medicine, Shanghai, China

^c^ Department of Orthopedics, Changzheng Hospital, Naval Medical University Shanghai, China

^d^ Research Institute of Plastic Surgery, Weifang Medical College, Shandong, China

^e^ Department of Plastic and Reconstructive Surgery, Shanghai Ninth People’s Hospital, Shanghai Jiao Tong University School of Medicine, Shanghai Key Laboratory of Tissue Engineering, Shanghai, China

^1^ These authors are equal to this work.

* Corresponding authors:

Dr. Huitang [Xia, Email: xiahuitang@163.com](mailto:Xia,%20Email:%20xiahuitang@163.com); Dr. Li Shen, Email: shenli@shchildren.com.cn; Dr. Depeng Meng, Email: [depengmeng@smmu.edu.cn](mailto:depengmeng@smmu.edu.cn); Dr. Chang Chen, Email: changchenc@tongji.edu.cn.

**Table 1. Primer sequences**

| **Gene** | **Primers** | |
| --- | --- | --- |
|  | **Forward (5’-3’)** | **Reverse (5’-3’)** |
| ACAN | TCGAGGACAGCGAGGCC | TCGAGGGTGTAGCGTGTAGAGA |
| Collagen II | AGCCTGGTGTCATGGGTTTC | GTCCCTTCTCACCAGCTTTGC |
| SOX9 | CCCCAACAGATCGCCTACAGT | GAGTTCTGGTGGTCGGTGTAGTC |
| Collagen I | CTGCCCAGAAGAATATGTATCACC | GAAGCAAAGTTTCCTCCAAGACC |
| OCN | CAAAGGTGCAGCCTTTGTGTC | TCACAGTCCGGATTGAGCTCA |
| RUNX2 | CCTTCCACTCTCAGTAAGAAGA | TAAGTAAAGGTGGCTGGATAGT |
| β-actin | ATCATGTTTGAGACCTTCAA | CATCTCTTGCTCGAAGTCCA |

**Table 2. The ICRS macroscopic scoring system**

| **Feature** | **Score** |
| --- | --- |
| Coverage |  |
| >75% fill | 4 |
| 50-75% fill | 3 |
| 25-50% fill | 2 |
| <25% fill | 1 |
| No fill | 0 |
| Neocartilage color |  |
| Normal | 4 |
| 25% yellow/brown | 3 |
| 50% yellow/brown | 2 |
| 75% yellow/brown | 1 |
| 100% yellow/brown | 0 |
| Defect margins |  |
| Invisible | 4 |
| 25% circumference visible | 3 |
| 50% circumference visible | 2 |
| 75% circumference visible | 1 |
| 100% circumference visible | 0 |
| Overall repair assessment |  |
| Grade I: normal | 12 |
| Grade II: nearly normal | 11-8 |
| Grade III: abnormal | 7-4 |
| Grade IV: severely abnormal | 3-1 |

**Table 3. O'Driscoll histological scoring system**

| **Feature** | **Score** |
| --- | --- |
| (a) Nature of the predominant tissue |  |
| 1 Cellular morphology | 4 |
| Hyaline articular cartilage | 2 |
| Incompletely differentiated mesenchyme | 0 |
| 2 Safranin-O staining of the matrix |  |
| Normal or nearly normal | 3 |
| Moderate | 2 |
| Slight | 1 |
| None | 0 |
| (b) Structural characteristics | 3 |
| 3 Surface regularity |  |
| Smooth and intact | 3 |
| Superficial horizontal lamination | 2 |
| Fissures-25 to 100 per cent of the thickness | 1 |
| Severe disruption | 0 |
| 4 Structural integrity |  |
| Normal | 2 |
| Slight disruption. including cysts | 1 |
| Severe disintegration | 0 |
| 5 Thickness |  |
| 100 per cent of normal adjacent cartilage | 2 |
| 50-100 per cent of normal cartilage | 1 |
| 0-50 per cent of normal cartilage | 0 |
| 6 Bonding to the adjacent cartilage |  |
| Bonded at both ends of graft | 2 |
| Bonded at one end, or partially at both ends | 1 |
| Not bonded | 0 |
| (c) Freedom from cellular changes of degeneration |  |
| 7 Hypocellularity |  |
| Normal cellularity | 3 |
| Slight hypocellularity | 2 |
| Moderate hypocellularity | 1 |
| Severe hypocellularity | 0 |
| 8 Chondrocyte clustering |  |
| No clusters | 2 |
| <25 per cent of the cells | 1 |
| 25-100 per cent of the cells | 0 |
| 9 Freedom from degenerative changes in adjacent cartilage |  |
| Normal cellularity. no clusters, normal staining | 3 |
| Normal cellularity, mild clusters, moderate staining | 2 |
| Mild or moderate hypocellularity, slight staining | 1 |
| Severe hypocellularity. poor or no staining | 0 |
